# Optimising Blood Test Monitoring Frequency for Inflammatory Bowel Disease Patients: Balancing Adherence, Comfort and Risk

**DOI:** 10.1155/cjgh/9272557

**Published:** 2026-10-08

**Authors:** Abhimati Ravikulan, James Irwin

**Affiliations:** ^1^ Department of Gastroenterology Palmerston North Hospital Palmerston North New Zealand

## Abstract

**Background:**

Inflammatory bowel disease (IBD) therapies can cause myelosuppression and hepatitis. The European Crohn’s and Colitis Organisation (ECCO) recommends 3‐monthly blood test monitoring; however, these recommendations are largely based on expert consensus, and real‐world evidence informing optimal long‐term monitoring intervals remains limited.

**Aim:**

To determine the incidence of medication‐related hepatitis and myelosuppression in a population‐based IBD cohort and estimate the laboratory monitoring burden required to detect clinically significant adverse events.

**Methods:**

A retrospective cohort study was performed using the Manawatū IBD incidence cohort. Patients diagnosed with IBD between 1 January 2011 and 31 December 2015 were followed longitudinally until 31 December 2022. Medication exposure, laboratory results, and clinical outcomes were recorded. Hepatitis was defined as alanine aminotransferase (ALT) > 2 × the upper limit of normal, while myelosuppression was defined as a neutrophil count < 1.0 × 10^9^/L. Patients were followed from diagnosis until the first outcome event or last recorded follow‐up. Medication exposure was analysed as a time‐varying variable using cox proportional hazards regression, and the duration of laboratory monitoring required to detect one adverse event was calculated.

**Results:**

Among 204 incident cases, 12 were excluded due to prediagnosis hepatitis or myelosuppression, leaving 192 patients for analysis. Twenty‐four hepatitis events and two myelosuppression events were observed during 1739 patient‐years of follow‐up. 6‐mercaptopurine (hazard ratio [HR] 39.2, *p* < 0.001), azathioprine (HR 6.26, *p* = 0.002), methotrexate (HR 9.94, *p* = 0.004) and lower mean corpuscular volume (HR 0.89, *p* = 0.001) were independently associated with hepatitis. Following the initial 6 months of therapy, one hepatitis event was detected for every 33 patient‐years of stable thiopurine exposure monitored with routine quarterly blood testing.

**Conclusion:**

Clinically significant hepatitis and myelosuppression were uncommon in this population‐based IBD cohort, particularly after the initial 6 months of therapy. These findings suggest that reduced laboratory monitoring frequency in carefully selected patients with established treatment stability warrants evaluation.

## 1. Introduction

Inflammatory bowel disease (IBD), encompassing Crohn’s disease and ulcerative colitis, is a chronic relapsing inflammatory condition of the gastrointestinal tract. It causes chronic diarrhoea, per rectal bleeding, abdominal pain, weight loss and can lead to bowel obstruction or bowel perforation. The management of IBD involves long‐term immunosuppressive therapy aimed at controlling inflammation, minimising symptoms and preventing complications. Medications commonly used include 5‐aminosalicylates (5‐ASA), thiopurines (azathioprine, 6‐mercaptopurine and thioguanine), methotrexate, biologic agents such as anti‐TNF therapies (infliximab, adalimumab), anti‐integrin therapy (vedolizumab), anti‐IL12/IL23 therapy (ustekinumab) and Janus Kinase 1 inhibitors (upadacitinib).

While these treatments are effective, they are also associated with potentially serious adverse effects, notably hepatotoxicity and myelosuppression. Thiopurines, in particular, carry a well‐established risk of dose‐independent hepatotoxicity (including cholestatic hepatitis, transaminitis and veno‐occlusive disease). Studies report hepatotoxicity in 3%–10% of patients and myelotoxicity in approximately 2%–5% of users, often linked to genetic polymorphisms affecting TPMT and NUDT15 activity [1–4]. Methotrexate is known to cause hepatic fibrosis with long‐term use, especially in patients with risk factors such as obesity or alcohol use [5]. Biologic medications are generally better tolerated but have been occasionally associated with liver injury and cytopenia. These events are rarer and are often confounded by concomitant medication use [6, 7].

Owing to these risks, clinical guidelines such as those from the European Crohn’s and Colitis Organisation (ECCO) and the British Society of Gastroenterology recommend regular monitoring of liver function and full blood counts, particularly in patients on thiopurines and methotrexate [8, 9]. ECCO suggests testing every 1–3 months after drug initiation, with some recommendations for reducing the frequency to three monthly once a stable regimen is achieved [9].

Despite these recommendations, the evidence underpinning long‐term monitoring intervals remains limited. Most guidance is based on expert opinion rather than prospective outcome data, and few studies have quantified the incidence of clinically significant adverse events after the initial treatment period. Routine three‐monthly testing also imposes substantial burdens on patients and healthcare systems, including inconvenience, reduced adherence, increased healthcare utilisation and financial cost. Whether this intensity of monitoring remains necessary after patients have demonstrated sustained treatment tolerance is uncertain.

## 2. Aim

To determine the incidence of medication‐related hepatitis and myelosuppression in a population‐based IBD cohort, identify factors associated with hepatotoxicity and quantify the laboratory monitoring burden required to detect clinically significant adverse events. These findings may help inform future studies evaluating risk‐stratified laboratory monitoring strategies for patients receiving long‐term immunomodulatory therapy.

## 3. Methods

This retrospective population‐based cohort study was conducted using the Manawatū IBD incidence cohort at Palmerston North Hospital, New Zealand, which includes all incident cases of Crohn’s disease, ulcerative colitis and IBD‐unclassified diagnosed between 1 January 2011 and 31 December 2015 [10]. Patients were followed longitudinally until 31 December 2022 or until their last recorded clinical follow‐up, whichever occurred first.

Basic demographic data, medication use (start date, stop date and dose) and the results of all blood tests performed were recorded. The medications used in the cohort included 5‐ASA (mesalazine, sulphasalazine and olsalazine), thiopurines (azathioprine, tioguanine and 6‐mercaptopurine), methotrexate and anti‐TNF biologic medication (adalimumab, infliximab).

The primary outcome was medication‐associated hepatitis, defined as an alanine aminotransferase (ALT) level exceeding twice the upper limit of normal (> 80 IU/L). The secondary outcome was myelosuppression, defined as a neutrophil count < 1.0 × 10^9^/L.

Patients with preexisting hepatitis or myelosuppression at the time of IBD diagnosis, as defined above, and patients with primary sclerosing cholangitis were excluded to minimise inclusion of preexisting liver disease unrelated to medication exposure. Overall cohort follow‐up commenced at the date of IBD diagnosis and continued until the first occurrence of the outcome of interest (hepatitis or myelosuppression) or the last available clinical follow‐up. Patients continued contributing follow‐up irrespective of medication status. Medication exposure was analysed separately from overall cohort follow‐up. For each medication, exposure time commenced on the date treatment was initiated and ended on the date treatment ceased. Consequently, an individual patient could contribute person‐time to multiple medication categories throughout follow‐up, including periods receiving no medication, periods receiving monotherapy and periods receiving combination therapy. The incidence of hepatitis and myelosuppression events was determined, and the duration of medication exposure required to detect one adverse event was subsequently calculated for each medication class.

## 4. Statistical Analysis

Statistical analyses were performed using R Statistical software (R Foundation for Statistical Computing, Vienna, Austria) [11].

Because medication exposure varied over time, cox proportional hazards regression with time‐varying covariates was used to evaluate factors associated with hepatitis. Owing to the very small number of myelosuppression events (*n* = 2), formal regression analysis was not undertaken for this outcome.

Candidate predictor variables included demographic characteristics (age, sex, ethnicity and IBD subtype), laboratory variables (TPMT activity, ASCA, albumin, C‐reactive protein, haemoglobin, lymphocyte count, eosinophil count, platelet count, mean corpuscular volume [MCV], mean corpuscular haemoglobin [MCH], 6‐thioguanine nucleotide [6‐TGN] concentration and 6‐methylmercaptopurine [6‐MMP] concentration), medication exposure and duration of follow‐up.

Medication exposure was modelled as a time‐varying covariate using 10‐day intervals. At each interval, medication status reflected the therapies actively prescribed during that period. Laboratory variables were similarly treated as time‐varying covariates, with the most recent available laboratory value assigned to each interval.

Accordingly, patients could contribute person‐time to more than one medication exposure category throughout follow‐up. For example, an individual patient could contribute observation time while receiving no medication, subsequently while receiving thiopurine monotherapy and later while receiving combination therapy with a biologic agent. This approach allowed medication exposure to reflect routine clinical practice and avoided assigning patients permanently to a single treatment group despite changes in therapy over time.

Variables with substantial missing data were excluded from regression modelling. Univariable cox proportional hazards regression was initially performed for each candidate predictor. Variables demonstrating an association with hepatitis at *p* < 0.20 were entered into the multivariable model. Backward elimination was subsequently performed until only independently associated variables remained. Potential interactions between variables retained in the final model were assessed.

A prespecified subgroup analysis was performed among patients receiving thiopurine therapy to evaluate the associations between TPMT activity, thiopurine metabolite concentrations (6‐TGN and 6‐MMP) and hepatitis.

Finally, medication‐specific person‐years of exposure were calculated to determine the duration of observation required to identify one hepatitis or myelosuppression event during both the treatment initiation period (≤ 6 months after treatment commencement) and the maintenance treatment period (> 6 months).

## 5. Results

A total of 204 incident cases of IBD were identified within the cohort. Eleven patients were excluded due to ALT levels exceeding twice the upper limit of normal prior to diagnosis of IBD, and one patient with prediagnosis neutropenia was also excluded. One hundred and ninety‐two patients were included in the analysis. The median duration of follow‐up was 9.29 years (interquartile range [IQR]: 7.33–10.74 years), providing 1739 patient‐years of data.

The cohort comprised 108 patients with Crohn’s disease (56.3%), 72 with ulcerative colitis (37.5%) and 12 with IBD‐unclassified (6.3%). Eighty‐three patients (43.2%) were male, and 109 (56.8%) were female. Most participants identified as New Zealand European (*n* = 176), followed by other ethnicities (*n* = 13) and Māori (*n* = 3).

32/192 patients (16.7%) remained untreated throughout follow‐up, with a median follow‐up duration of 10.43 years (IQR 5.95–11.93 years). A further 97 patients (50.1%) spent a median of 2.49 years (IQR 0.37–6.26) taking no medication.

During follow‐up, 146 patients (76.0%) received a 5‐ASA, with a median exposure duration of 4.48 years (IQR 0.93–8.82 years); 75 patients (39.1%) received a thiopurine medication, with a median exposure duration of 1.67 years (IQR 0.27–5.17 years); 15 patients (7.8%) received methotrexate, with a median exposure duration of 1.09 years (IQR 0.26–5.28 years); and 31 patients (16.1%) received an anti‐TNF biologic agent, with a median exposure duration of 2.14 years (IQR 1.10–5.95 years).

Medication‐specific person‐years of exposure, rather than the number of patients ever exposed to a medication, are presented in Table 1. Twenty‐four hepatitis events and two episodes of myelosuppression were identified during follow‐up. Because only two myelosuppression events occurred, meaningful regression analysis was not possible, and subsequent analyses focused on hepatitis.

**TABLE 1:** Testing data.

	No medication	5‐ASA	Thiopurine	Methotrexate	Biologic
Patients contributing data	129	146	75	15	31
Total follow‐up (patient‐years)	822.2	755.1	269.8	42.9	146.1
Per patient follow‐up while taking this medication (median years, IQR)	1.6 (0.56–5.62)	4.48 (0.93–8.82)	1.67 (0.27 – 5.17)	1.09 (0.26–5.28)	2.14 (1.10–5.95)
Total tests	692	1257	905	86	358
Tests per year per patient	1.21	1.77	3.92	3.4	3.2
Hepatitis events within 6 months medication initiation period	3	3	7	1	1
Testing duration required, within medication initiation period, to detect one hepatitis event (patient years)	23.9	21.8	4.5	3.9	13.8
Hepatitis events following medication initiation period	2	9	6	1	3
Testing duration required, following medication initiation period, to detect one hepatitis event (patient years)	250	71.9	33.3	21.3	32.7

*Note:* Frequency of testing and frequency of hepatitis events stratified by medication use.

The longitudinal trajectory of ALT measurements for individual patients is shown in Figure 1, illustrating the timing of hepatitis events during medication exposure. Among patients who developed hepatitis while receiving treatment, subsequent ALT trajectories were examined to determine whether liver enzyme abnormalities resolved following continuation or cessation of therapy (Figures 2 and 3). Twelve patients discontinued the implicated medication at the time hepatitis occurred, whereas the remaining 12 continued treatment. ALT concentrations subsequently normalised in the majority of patients, regardless of whether treatment was discontinued or continued.

**FIGURE 1:**
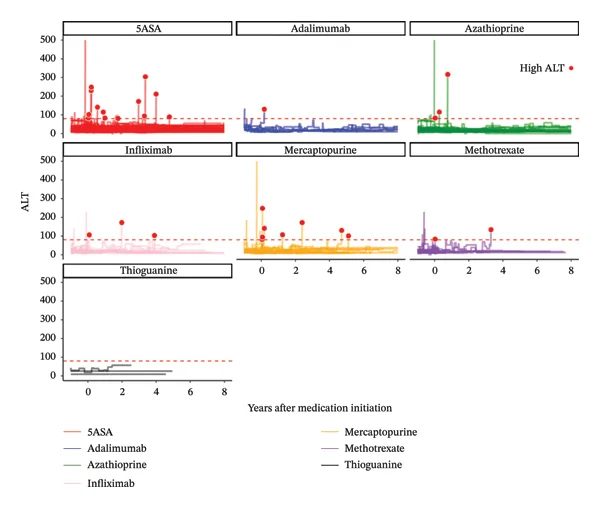
Each point represents an individual ALT measurement. The dashed horizontal line represents the hepatitis threshold (ALT > 2 × ULN). Patients were censored at the first hepatitis event or end of follow‐up.

**FIGURE 2:**
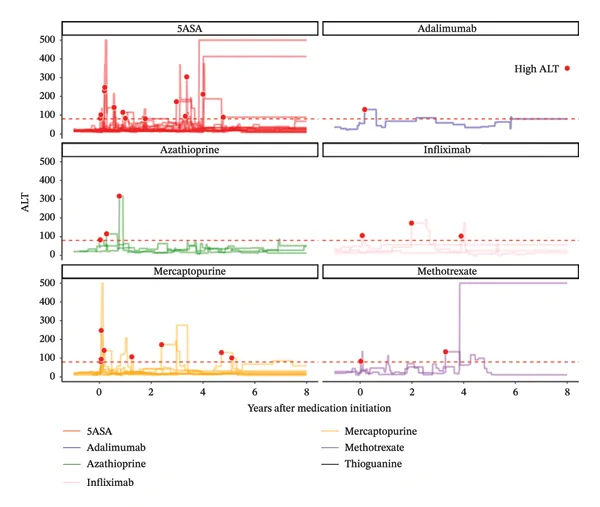
Subsequent trajectory of ALT values for all patients who had a hepatitis event, demonstrating normalisation of ALT values for most patients.

**FIGURE 3:**
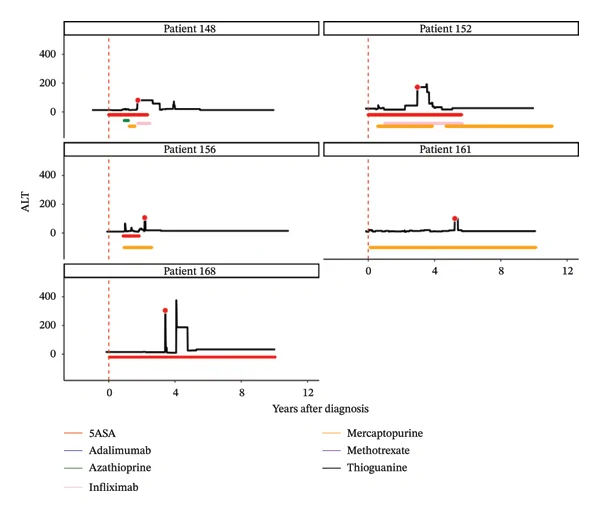
Medication use and subsequent trajectory of ALT values for selected patients who had a hepatitis event, demonstrating continuation or cessation of medications at the time of the event.

Medication‐specific laboratory testing frequency and hepatitis incidence are summarised in Table 1. Among patients receiving thiopurines, routine laboratory monitoring occurred approximately every 3 months. Seven hepatitis events occurred during the first 6 months of thiopurine therapy, whereas six events occurred after completion of the initial 6‐month treatment period.

Beyond the first 6 months of treatment, one hepatitis event was detected for every 33 patient‐years of stable thiopurine exposure monitored with routine quarterly blood testing. Corresponding estimates for other medication classes are presented in Table 1. Multivariable cox proportional hazards regression identified four variables independently associated with hepatitis (Table 2): azathioprine use (HR 6.25, *p* = 0.002), 6‐mercaptopurine use (HR 39.24, *p* < 0.001), methotrexate use (HR 9.94, *p* = 0.004) and lower MCV (HR 0.89, *p* = 0.001).

**TABLE 2:** Final cox regression model correlating predictor variables with hepatitis events.

	Coef	Hazard ratio	95% confidence interval	Se (coef)	*z*	*p* value
Azathiopurine	1.83	6.26	(1.97–19.84)	0.59	3.11	0.002
Mercaptopurine	3.67	39.24	(14.66–105.05)	0.50	7.30	< 0.001
Methotrexate	2.30	9.94	(2.06–48.05)	0.80	2.86	0.004
MCV	−0.12	0.89	(0.83–0.95)	0.04	−3.29	0.001

Supporting Table S1 summarises the results of the univariable analyses used for multivariable model selection.

Among patients receiving thiopurine therapy, lower MCV remained independently associated with hepatitis (Table 3). Male sex (*p* = 0.019) and higher 6‐MMP concentrations (*p* < 0.001) were also independently associated with hepatitis within this subgroup.

**TABLE 3:** Final cox regression model correlating predictor variables with hepatitis events—analysis limited to patients taking thiopurine medication.

	Coef	Hazard ratio	95% confidence interval	Se (coef)	*z*	*p* value
Gender (male)	1.65	5.21	(1.32–20.59)	0.70	2.36	0.019
MCV	−0.15	0.86	(0.76–0.97)	0.06	−2.41	0.016
MMP	0.00	1.00	(1.00–1.00)	0.00	3.65	< 0.001

## 6. Discussion

In this population‐based IBD cohort, clinically significant hepatitis and myelosuppression were uncommon during long‐term follow‐up, particularly after the initial 6 months of immunomodulatory therapy. These findings are consistent with previous studies demonstrating that thiopurine‐associated hepatotoxicity most commonly occurs early after treatment initiation, with the majority of liver enzyme abnormalities developing within the first few months of therapy [1, 3, 12].

A novel finding of this study is the quantification of the laboratory monitoring burden required to detect clinically significant hepatotoxicity. Among patients receiving thiopurine therapy, one hepatitis event was detected for every 33 patient‐years of stable thiopurine exposure monitored with routine quarterly blood testing. Although similar trends were observed for methotrexate and anti‐TNF therapy, the smaller number of exposed patients limits interpretation for these treatment groups.

Importantly, many patients who developed hepatitis subsequently demonstrated normalisation of ALT concentrations, including patients who continued their prescribed therapy. This observation suggests that not all late ALT elevations necessarily represent clinically significant or progressive hepatotoxicity requiring treatment discontinuation. However, the retrospective design of this study precludes conclusions regarding the clinical consequences of delayed recognition of these abnormalities.

The very low incidence of myelosuppression observed in our cohort (two events over 1739 patient‐years of follow‐up) prevented meaningful statistical analysis. Nevertheless, this finding is consistent with previous population‐based studies reporting that clinically significant thiopurine‐associated myelotoxicity occurs predominantly during the early phase of treatment [2, 13]. For example, Gisbert et al. demonstrated that most cases of azathioprine‐induced myelotoxicity occurred within the first 8 weeks of therapy [2].

An additional finding of interest was the independent association between lower MCV and hepatitis among patients receiving thiopurines. To our knowledge, this association has not previously been reported. Dujardin et al. demonstrated that increasing MCV correlates with therapeutic 6‐TGN concentrations but is an unreliable surrogate marker of clinical remission [14]. One possible explanation for our findings is that patients with lower MCV may exhibit a different thiopurine metabolite profile that predisposes to hepatotoxicity rather than bone marrow effects. Interestingly, the association between lower MCV and hepatitis remained independent of both 6‐TGN and 6‐MMP concentrations, suggesting that this relationship is unlikely to be explained solely by conventional thiopurine metabolite patterns. Confirmation of this observation in larger prospective cohorts is warranted before MCV can be considered a clinically useful predictor of hepatotoxicity.

The observed association between elevated 6‐MMP concentrations and hepatitis is consistent with previous reports and supports the recognised role of preferential methylation in thiopurine‐induced liver injury [15].

Similarly, the association between male sex and hepatitis among thiopurine users is supported by previous work. Wong et al. identified male sex, increasing body mass index and elevated early 6‐MMP concentrations as independent predictors of thiopurine‐induced hepatotoxicity [16]. However, other prospective studies have not consistently demonstrated this relationship [17], suggesting that further investigation is required before sex can be considered a reliable risk marker.

The principal strength of this study is its population‐based incidence cohort with prolonged follow‐up, providing 1739 patient‐years of observation and detailed longitudinal medication and laboratory data. Unlike studies restricted to tertiary referral populations, this cohort reflects routine clinical practice and therefore provides clinically relevant estimates of adverse event frequency during long‐term therapy. Furthermore, by analysing medication exposure as a time‐varying covariate, the study more accurately reflects real‐world treatment patterns, where patients frequently commence, discontinue and combine therapies over time.

Current international guidelines adopt a cautious approach to laboratory monitoring. ECCO recommends intensive monitoring during treatment initiation followed by testing every two to 3 months during maintenance therapy [18], while the British Society of Gastroenterology recommends three‐monthly full blood count and liver function monitoring during ongoing immunosuppressive treatment [9]. These recommendations are largely based on expert consensus rather than direct evidence defining optimal long‐term monitoring intervals.

Our findings suggest that the diagnostic yield of routine laboratory monitoring decreases substantially once patients have demonstrated treatment stability during the first 6 months of therapy. However, this study was not designed to determine whether extending monitoring intervals is clinically equivalent or superior to current practice. Consequently, although these data support further evaluation of risk‐stratified monitoring strategies, they should not be interpreted as sufficient evidence to recommend immediate changes to existing clinical guidelines.

This study has several limitations. First, its retrospective design introduces the possibility of incomplete ascertainment of laboratory abnormalities or adverse events occurring outside the regional health service. Second, newer therapies such as tofacitinib, upadacitinib, vedolizumab and ustekinumab were not routinely available during the study period and therefore could not be evaluated. Third, the relatively small number of patients receiving methotrexate or anti‐TNF therapy limits the precision of medication‐specific estimates for these agents. Finally, only 24 hepatitis events occurred during follow‐up. Although a prespecified modelling strategy and time‐varying cox regression were used to minimise bias, the relatively small number of outcome events may limit model stability and increase the possibility of overfitting. Consequently, the identified predictors should be interpreted as exploratory and require external validation in larger prospective cohorts before being incorporated into clinical decision‐making.

## 7. Conclusion

Clinically significant hepatitis and myelosuppression were uncommon among patients receiving long‐term immunomodulatory therapy for IBD, particularly after the initial 6 months of treatment. Among patients receiving stable thiopurine therapy beyond this period, one hepatitis event was detected for every 33 patient‐years of routine quarterly blood test monitoring.

These findings suggest that the diagnostic yield of routine laboratory monitoring decreases once treatment stability has been established. While the results support further evaluation of risk‐stratified monitoring strategies in carefully selected patients, they should not be interpreted as sufficient evidence to justify immediate changes to current guideline‐directed monitoring intervals.

Prospective, multicentre studies are now required to determine whether extending monitoring intervals in low‐risk patients can safely reduce healthcare utilisation while maintaining patient safety and treatment outcomes.

## Funding

No funding was used in this study.

## Conflicts of Interest

The authors declare no conflicts of interest.

## Supporting Information

Additional supporting information can be found online in the Supporting Information section.

## Supporting information


**Supporting Information** Table S1: Univariate cox regression correlating predictor variables with hepatitis events.

## Data Availability

The data that support the findings of this study are available on request from the corresponding author. The data are not publicly available due to privacy or ethical restrictions.
