# Supplementary material for: Optimising Blood Test Monitoring Frequency for Inflammatory Bowel Disease Patients: Balancing Adherence, Comfort and Risk
Source: Can J Gastroenterol Hepatol. 2026 Oct 8;2026:9272557. doi: 10.1155/cjgh/9272557 (PMC13646887; doi:10.1155/cjgh/9272557)
Supplement: Supplementary file 1 [file CJGH-2026-9272557-s001.docx]

| Variable | coef | Hazard ratio | 95% Confidence Interval | se(coef) | z | P-value |
| --- | --- | --- | --- | --- | --- | --- |
| Mercaptopurine use | 2.7343 | 15.3993 | (6.83 - 34.71) | 0.4146 | 6.5946 | 0.0000 |
| 6-MMP level | 0.0002 | 1.0002 | (1.00 - 1.00) | 0.0001 | 3.6264 | 0.0003 |
| Infliximab use | 1.6899 | 5.4187 | (2.02 - 14.54) | 0.5037 | 3.355 | 0.0008 |
| Haemoglobin level | -0.0421 | 0.9588 | (0.93 - 0.98) | 0.0132 | -3.1836 | 0.0015 |
| ALBUMIN level | -0.087 | 0.9166 | (0.85 - 0.98) | 0.0365 | -2.3862 | 0.017 |
| SixTG level | 0.0025 | 1.0025 | (1.00 - 1.00) | 0.0011 | 2.3383 | 0.0194 |
| Eosinophil cound | -3.6095 | 0.0271 | (0.00 - 1.00) | 1.8424 | -1.9591 | 0.0501 |
| PLT level | 0.0042 | 1.0042 | (1.00 - 1.01) | 0.0022 | 1.9331 | 0.0532 |
| Methotrexate | 1.3858 | 3.9979 | (0.94 - 17.02) | 0.7392 | 1.8748 | 0.0608 |
| MCV | -0.0744 | 0.9283 | (0.86 - 1.01) | 0.0406 | -1.8339 | 0.0667 |
| Lymphocyte count | -0.4321 | 0.6491 | (0.36 - 1.15) | 0.294 | -1.4699 | 0.1416 |
| Azathioprine use | 0.6465 | 1.9088 | (0.71 - 5.11) | 0.5029 | 1.2856 | 0.1986 |
| Duration of follow-up | -0.1111 | 0.8948 | (0.74 - 1.09) | 0.0985 | -1.1282 | 0.2592 |
| TPMT level | 0.0043 | 1.0043 | (1.00 - 1.01) | 0.004 | 1.0801 | 0.2801 |
| DIAGNOSIS: Ulcerative_Colitis | -0.1473 | 0.863 | (0.37 - 2.02) | 0.4333 | -0.3399 | 0.7339 |
| Male gender | 0.1339 | 1.1433 | (0.51 - 2.55) | 0.4097 | 0.3268 | 0.7438 |
| MCH | -0.0306 | 0.9698 | (0.79 - 1.19) | 0.1055 | -0.2903 | 0.7716 |
| age | -0.0002 | 0.9998 | (1.00 - 1.00) | 0.0018 | -0.1119 | 0.9109 |
| CRP level | 0.0005 | 1.0005 | (0.99 - 1.01) | 0.0054 | 0.0991 | 0.9211 |
| ASCA level | -0.0023 | 0.9977 | (0.95 - 1.04) | 0.0234 | -0.0974 | 0.9224 |
| FiveASA use. | 0.0328 | 1.0334 | (0.46 - 2.31) | 0.4112 | 0.0798 | 0.9364 |
| Adalimumab use | -16.0782 | 0 | (0.00 - Inf) | 2828.2715 | -0.0057 | 0.9955 |
| Thioguanine use | -15.0319 | 0 | (0.00 - Inf) | 2890.8414 | -0.0052 | 0.9959 |

Table S1: Univariate cox regression correlating predictor variables with hepatitis events.
